# Supplementary material for: Identification of the Key microRNAs and miRNA-mRNA Interaction Networks during the Ovarian Development of Hens
Source: Animals (Basel). 2020 Sep 17;10(9):1680. doi: 10.3390/ani10091680 (PMC7552605; doi:10.3390/ani10091680)
Supplement: Supplementary file 1 [file animals-10-01680-s001.pdf]

Article

Identification of the Key microRNAs and miRNA-mRNA Interaction Networks During the Ovarian Development of Hens

Jing Li †, Chong Li †, Qi Li, Wen-Ting Li, Hong Li, Guo-Xi Li, Xiang-Tao Kang, Xiao-Jun Liu and Ya-Dong Tian *

College of Animal Science and Technology, Henan Agricultural University, Zhengzhou 450046, China; lijingbj_87@126.com (J.L.); lc0924ll@163.com (C.L.); m18838939701@163.com (Q.L.); liwenting_5959@hotmail.com (W.-T.L.); lihong19871202@163.com (H.L.); liguoxi0914@126.com (G.-X.L.); xtkang2001@263.net (X.-T.K.); xjliu2008@hotmail.com (X.-J.L.)

* Correspondence: tianyadong@henau.edu.cn

† These two authors contributed equally to this work.

Received: 27 July 2020; Accepted: 15 September 2020; Published: date

Supplementary Material

Table 1. The list of the interaction network, the expression levels and Pearson's correlation coefficient of DE miRNAs and DE mRNAs.

**sRNA**	**Expression Level (TPM)**				**Transcript Id**	**Gene Id**	**Gene Name**	**Expression Level (FPKM)**				**Correlatio**
	15W	20W	30W	68W				15W	20W	30W	68W	
gga-miR-1560-3p	3.253	6.030	4.295	2.565	ENSGALT00000087050	ENSGALG00000005902	RAB7A	17.832	0.031	6.674	0.077	-0.324
gga-miR-143-3p	25118.987	49390.256	87681.664	32277.275	ENSGALT00000069072	ENSGALG00000041760	CLTCL1	2.189	0.000	1.321	1.252	-0.268
gga-miR-7472-5p	0.054	0.264	0.466	0.000	ENSGALT00000066785	ENSGALG00000014582	CADM1	6.810	2.342	0.000	0.000	-0.394
gga-miR-7472-5p	0.054	0.264	0.466	0.000	ENSGALT00000033172	ENSGALG00000008121	CYP17A1	722.987	675.854	233.756	239.151	-0.236
gga-miR-133a-3p	107.574	99.409	202.785	80.977	ENSGALT00000093261	ENSGALG00000001171	SEMA6B	2.182	0.013	0.000	0.000	-0.187
gga-miR-133a-3p	107.574	99.409	202.785	80.977	ENSGALT00000091328	ENSGALG00000008885	PDE1A	1.907	0.005	0.006	0.006	-0.185
gga-miR-133a-3p	107.574	99.409	202.785	80.977	ENSGALT00000099646	ENSGALG00000007116	CABP1	0.457	0.197	0.000	0.258	-0.722
gga-miR-133a-3p	107.574	99.409	202.785	80.977	ENSGALT00000048408	ENSGALG00000006895	CEP131	1.062	0.001	0.000	1.493	-0.632
gga-miR-133a-3p	107.574	99.409	202.785	80.977	ENSGALT00000107742	ENSGALG00000000374	RNPEP	3.370	0.000	0.000	0.000	-0.185
gga-miR-210b-5p	11.042	11.594	22.074	7.613	ENSGALT00000070461	ENSGALG00000004171	RNF111	0.509	0.298	0.000	0.301	-0.796
gga-miR-210b-5p	11.042	11.594	22.074	7.613	ENSGALT00000087758	ENSGALG00000000652	ACOT7	0.639	1.106	0.000	2.724	-0.818
gga-miR-133c-3p	16.707	15.505	33.699	9.452	ENSGALT00000048408	ENSGALG00000006895	CEP131	1.062	0.001	0.000	1.493	-0.689
gga-miR-133c-3p	16.707	15.505	33.699	9.452	ENSGALT00000107742	ENSGALG00000000374	RNPEP	3.370	0.000	0.000	0.000	-0.137
gga-miR-133c-3p	16.707	15.505	33.699	9.452	ENSGALT00000099646	ENSGALG00000007116	CABP1	0.457	0.197	0.000	0.258	-0.690
gga-miR-133c-3p	16.707	15.505	33.699	9.452	ENSGALT00000012517	ENSGALG00000007720	AIFM3	0.453	0.226	0.000	0.319	-0.793
gga-miR-133c-3p	16.707	15.505	33.699	9.452	ENSGALT00000093261	ENSGALG00000001171	SEMA6B	2.182	0.013	0.000	0.000	-0.138
gga-miR-143-5p	326.004	491.847	823.909	272.415	ENSGALT00000048408	ENSGALG00000006895	CEP131	1.062	0.001	0.000	1.493	-0.831
gga-miR-143-5p	326.004	491.847	823.909	272.415	ENSGALT00000009304	ENSGALG00000030677	ABCG2	12.705	13.497	0.000	0.000	-0.312
gga-miR-143-5p	326.004	491.847	823.909	272.415	ENSGALT00000074641	ENSGALG00000006851	-	2.618	1.886	0.019	1.297	-0.775
gga-miR-143-5p	326.004	491.847	823.909	272.415	ENSGALT00000027488	ENSGALG00000017018	INTS6	2.543	0.274	0.000	1.508	-0.794
gga-miR-143-5p	326.004	491.847	823.909	272.415	ENSGALT00000100689	ENSGALG00000010733	CEP170	0.275	0.000	0.000	2.014	-0.632
gga-miR-143-5p	326.004	491.847	823.909	272.415	ENSGALT00000096177	ENSGALG00000041423	MAP7D3	5.262	2.400	0.551	4.255	-0.940
gga-miR-143-5p	326.004	491.847	823.909	272.415	ENSGALT00000016815	ENSGALG00000010331	MME	42.012	32.170	13.326	10.675	-0.319
gga-miR-143-5p	326.004	491.847	823.909	272.415	ENSGALT00000095588	ENSGALG00000006704	VPS8	1.563	1.554	0.000	1.014	-0.779

gga-miR-147	8.794	11.399	21.487	8.411	ENSGALT00000078929	ENSGALG00000000336	PPP1R12B	8.372	3.379	1.573	2.312	-0.540
gga-miR-147	8.794	11.399	21.487	8.411	ENSGALT00000104695	ENSGALG00000047393	-	1.376	0.900	0.000	0.477	-0.751
gga-miR-2188-5p	36.772	53.518	59.863	48.895	ENSGALT00000064808	ENSGALG00000039742	CYP21	22.317	18.391	2.432	10.416	-0.795
gga-miR-2188-5p	36.772	53.518	59.863	48.895	ENSGALT00000081827	ENSGALG00000020742	PPRC1	0.762	0.165	0.000	0.186	-0.968
gga-miR-365-2-5p	1.403	2.721	3.552	1.889	ENSGALT00000095588	ENSGALG00000006704	VPS8	1.563	1.554	0.000	1.014	-0.739
gga-miR-365-2-5p	1.403	2.721	3.552	1.889	ENSGALT00000093261	ENSGALG00000001171	SEMA6B	2.182	0.013	0.000	0.000	-0.696
gga-miR-365-2-5p	1.403	2.721	3.552	1.889	ENSGALT00000081827	ENSGALG00000020742	PPRC1	0.762	0.165	0.000	0.186	-0.838
gga-miR-365-2-5p	1.403	2.721	3.552	1.889	ENSGALT00000005128	ENSGALG00000003242	STAR	298.770	154.884	31.693	48.914	-0.665
gga-miR-365-2-5p	1.403	2.721	3.552	1.889	ENSGALT00000071400	ENSGALG00000010049	PTPRB	2.151	0.001	0.000	0.068	-0.715
gga-miR-365-2-5p	1.403	2.721	3.552	1.889	ENSGALT00000012517	ENSGALG00000007720	AIFM3	0.453	0.226	0.000	0.319	-0.986
gga-miR-365-2-5p	1.403	2.721	3.552	1.889	ENSGALT00000093800	ENSGALG00000010664	KIF26B	0.516	0.007	0.006	0.003	-0.693
gga-miR-193a-5p	11.947	13.010	17.887	13.712	ENSGALT00000106245	ENSGALG00000015226	SPIDR	1.548	1.039	0.000	0.534	-0.924
gga-miR-193a-5p	11.947	13.010	17.887	13.712	ENSGALT00000002237	ENSGALG00000001475	STMN1	29.135	11.631	0.000	10.851	-0.868
gga-miR-193b-3p	5.495	5.774	9.309	4.190	ENSGALT00000099612	ENSGALG00000054034	TNKS1BP1	1.927	0.000	0.000	0.000	-0.212
gga-miR-193b-3p	5.495	5.774	9.309	4.190	ENSGALT00000017675	ENSGALG00000010874	UNC79	5.329	3.249	0.939	2.272	-0.551
gga-miR-193b-3p	5.495	5.774	9.309	4.190	ENSGALT00000064808	ENSGALG00000039742	CYP21	22.317	18.391	2.432	10.416	-0.630
gga-miR-24-3p	913.412	963.960	1678.040	833.186	ENSGALT00000001662	ENSGALG00000001108	MIER2	3.156	0.000	0.000	0.000	-0.313
gga-miR-24-3p	913.412	963.960	1678.040	833.186	ENSGALT00000098722	ENSGALG00000009915	EML4	1.337	0.777	0.000	0.530	-0.751
gga-miR-24-3p	913.412	963.960	1678.040	833.186	ENSGALT00000059290	ENSGALG00000010879	FGGY	0.529	0.256	0.009	0.672	-0.879
gga-miR-24-3p	913.412	963.960	1678.040	833.186	ENSGALT00000044013	ENSGALG00000004671	ANKRD27	2.304	1.178	0.000	0.923	-0.741
gga-miR-24-3p	913.412	963.960	1678.040	833.186	ENSGALT00000100689	ENSGALG00000010733	CEP170	0.275	0.000	0.000	2.014	-0.512
gga-miR-365-1-5p	0.581	0.457	1.890	1.661	ENSGALT00000093800	ENSGALG00000010664	KIF26B	0.516	0.007	0.006	0.003	-0.518
gga-miR-365-1-5p	0.581	0.457	1.890	1.661	ENSGALT00000095588	ENSGALG00000006704	VPS8	1.563	1.554	0.000	1.014	-0.889
gga-miR-365-1-5p	0.581	0.457	1.890	1.661	ENSGALT00000005128	ENSGALG00000003242	STAR	298.770	154.884	31.693	48.914	-0.841
gga-miR-365-1-5p	0.581	0.457	1.890	1.661	ENSGALT00000071400	ENSGALG00000010049	PTPRB	2.151	0.001	0.000	0.068	-0.506
gga-miR-365-1-5p	0.581	0.457	1.890	1.661	ENSGALT00000093261	ENSGALG00000001171	SEMA6B	2.182	0.013	0.000	0.000	-0.520
gga-miR-34a-5p	16.395	10.628	29.991	23.554	ENSGALT00000091480	ENSGALG00000015787	RNGTT	11.812	6.155	1.273	8.937	-0.527
gga-miR-34a-5p	16.395	10.628	29.991	23.554	ENSGALT00000011022	ENSGALG00000006818	-	0.400	0.001	0.001	0.001	-0.296

gga-miR-34a-5p	16.395	10.628	29.991	23.554	ENSGALT00000101371	ENSGALG00000012934	BICD1	3.700	1.141	0.002	0.863	-0.541
gga-miR-146a-3p	13.579	15.887	20.732	28.434	ENSGALT00000070917	ENSGALG00000029917	SGCA	0.138	0.169	0.000	0.053	-0.662
gga-miR-146a-3p	13.579	15.887	20.732	28.434	ENSGALT00000101295	ENSGALG00000005646	TSC2	0.486	0.919	0.000	0.000	-0.737
gga-miR-146a-3p	13.579	15.887	20.732	28.434	ENSGALT00000101350	ENSGALG00000037370	NAV1	0.241	0.000	0.000	0.016	-0.570
gga-miR-146a-3p	13.579	15.887	20.732	28.434	ENSGALT00000075830	ENSGALG00000008368	RAD18	0.672	0.184	0.000	0.553	-0.012
gga-miR-146a-3p	13.579	15.887	20.732	28.434	ENSGALT00000093261	ENSGALG00000001171	SEMA6B	2.182	0.013	0.000	0.000	-0.621
gga-miR-146a-3p	13.579	15.887	20.732	28.434	ENSGALT00000095588	ENSGALG00000006704	VPS8	1.563	1.554	0.000	1.014	-0.446
gga-miR-146a-3p	13.579	15.887	20.732	28.434	ENSGALT00000093800	ENSGALG00000010664	KIF26B	0.516	0.007	0.006	0.003	-0.622
gga-miR-146a-3p	13.579	15.887	20.732	28.434	ENSGALT00000001662	ENSGALG00000001108	MIER2	3.156	0.000	0.000	0.000	-0.617
gga-miR-146a-3p	13.579	15.887	20.732	28.434	ENSGALT00000099646	ENSGALG00000007116	CABP1	0.457	0.197	0.000	0.258	-0.339
gga-miR-146a-3p	13.579	15.887	20.732	28.434	ENSGALT00000073855	ENSGALG00000014795	TGIF1	7.612	5.591	0.119	2.800	-0.662
gga-miR-146a-3p	13.579	15.887	20.732	28.434	ENSGALT00000102768	ENSGALG00000002643	-	1.676	0.057	0.000	1.762	-0.263
gga-miR-302b-3p	22.298	0.305	2.840	3.852	ENSGALT00000084083	ENSGALG00000043379	RPL14	165.021	175.280	31.810	147.775	-0.291
gga-miR-146b-5p	8117.249	8015.714	17158.741	8507.245	ENSGALT00000026780	ENSGALG00000016592	PRPS2	0.778	1.435	0.082	1.225	-0.888
gga-miR-34a-5p	16.395	10.628	29.991	23.554	ENSGALT00000093630	ENSGALG00000043576	AGAP3	0.000	0.406	0.000	0.000	-0.752
gga-miR-34a-5p	16.395	10.628	29.991	23.554	ENSGALT00000035097	ENSGALG00000021647	NRTN	0.204	1.902	0.044	3.635	-0.141
gga-miR-34a-5p	16.395	10.628	29.991	23.554	ENSGALT00000069310	ENSGALG00000035602	ZNF536	0.008	0.675	0.005	0.006	-0.755
gga-miR-34a-5p	16.395	10.628	29.991	23.554	ENSGALT00000103998	ENSGALG00000034475	EIF3G	0.000	7.082	0.000	11.292	-0.205
gga-miR-34a-5p	16.395	10.628	29.991	23.554	ENSGALT00000054394	ENSGALG00000011662	NUP205	4.060	7.402	0.453	1.066	-0.962
gga-miR-34a-5p	16.395	10.628	29.991	23.554	ENSGALT00000095537	ENSGALG00000028561	SZT2	0.000	0.646	0.000	0.497	-0.524
gga-miR-365-1-5p	0.581	0.457	1.890	1.661	ENSGALT00000083042	ENSGALG00000040369	PFKFB3	0.009	1.339	0.014	0.579	-0.449
gga-miR-146a-3p	13.579	15.887	20.732	28.434	ENSGALT00000107299	ENSGALG00000037869	-	0.396	0.000	0.308	0.000	-0.511
gga-miR-365-1-5p	0.581	0.457	1.890	1.661	ENSGALT00000037720	ENSGALG00000011171	TBP	4.296	1.744	4.104	0.014	-0.129
gga-miR-6606-5p	1.384	2.794	0.925	2.184	ENSGALT00000001353	ENSGALG00000000919	PIGR	3.927	2.400	2.500	0.181	-0.405
gga-miR-6606-5p	1.384	2.794	0.925	2.184	ENSGALT00000097297	ENSGALG00000033170	GMDS	1.229	0.310	0.014	0.036	-0.166
gga-miR-6606-5p	1.384	2.794	0.925	2.184	ENSGALT00000096958	ENSGALG00000003741	MYO18A	0.561	0.498	0.582	0.000	-0.412
gga-miR-34a-5p	16.395	10.628	29.991	23.554	ENSGALT00000073682	ENSGALG00000002347	DIPK2A	0.312	0.000	0.111	0.000	-0.020
gga-miR-365-1-5p	0.581	0.457	1.890	1.661	ENSGALT00000061072	ENSGALG00000016391	CNKSR2	0.063	0.361	0.076	0.000	-0.646

gga-miR-365-1-5p	0.581	0.457	1.890	1.661	ENSGALT00000055413	ENSGALG00000011570	ILF2	0.107	1.735	0.556	0.000	-0.484
gga-miR-146a-3p	13.579	15.887	20.732	28.434	ENSGALT00000094599	ENSGALG00000054458	AKT2	0.050	0.425	0.372	0.000	-0.351
gga-miR-146a-3p	13.579	15.887	20.732	28.434	ENSGALT00000058144	ENSGALG00000032184	CSPG5	0.031	0.319	0.163	0.000	-0.424
gga-miR-146a-3p	13.579	15.887	20.732	28.434	ENSGALT00000097817	ENSGALG00000051144	EVI5L	0.419	2.392	1.284	0.000	-0.489
gga-miR-146a-3p	13.579	15.887	20.732	28.434	ENSGALT00000093630	ENSGALG00000043576	AGAP3	0.000	0.406	0.000	0.000	-0.383
gga-miR-204	173.788	85.174	21.500	43.574	ENSGALT00000007773	ENSGALG00000004869	ISCU	21.898	30.649	31.628	10.988	-0.069
gga-miR-449c-5p	10.423	3.526	1.891	1.095	ENSGALT00000103998	ENSGALG00000034475	EIF3G	0.000	7.082	0.000	11.292	-0.569
gga-miR-449c-5p	10.423	3.526	1.891	1.095	ENSGALT00000094484	ENSGALG00000014155	OGDH	0.000	0.159	0.132	0.166	-0.954
gga-miR-449c-5p	10.423	3.526	1.891	1.095	ENSGALT00000081517	ENSGALG00000001331	ST14	0.003	1.783	0.779	1.383	-0.750
gga-miR-449c-5p	10.423	3.526	1.891	1.095	ENSGALT00000105774	ENSGALG00000013462	STX17	0.032	1.541	0.506	0.515	-0.456
gga-miR-449c-5p	10.423	3.526	1.891	1.095	ENSGALT00000093630	ENSGALG00000043576	AGAP3	0.000	0.406	0.000	0.000	-0.111
gga-miR-449c-5p	10.423	3.526	1.891	1.095	ENSGALT00000107116	ENSGALG00000003601	ATP2B4	0.000	0.662	1.826	1.436	-0.893
gga-miR-449c-5p	10.423	3.526	1.891	1.095	ENSGALT00000011727	ENSGALG00000007247	PXN	0.001	1.756	3.481	0.823	-0.629
gga-miR-449c-5p	10.423	3.526	1.891	1.095	ENSGALT00000054103	ENSGALG00000001157	DENND1A	0.006	2.860	0.016	0.015	-0.114
gga-miR-449c-5p	10.423	3.526	1.891	1.095	ENSGALT00000079430	ENSGALG00000033061	CAMK2B	0.000	0.366	0.278	0.124	-0.628
gga-miR-1720-5p	10.651	5.238	1.120	1.726	ENSGALT00000081517	ENSGALG00000001331	ST14	0.003	1.783	0.779	1.383	-0.576
gga-miR-1720-5p	10.651	5.238	1.120	1.726	ENSGALT00000095819	ENSGALG00000040241	NTRK3	0.000	0.780	1.302	0.000	-0.503
gga-miR-449b-5p	7.996	4.869	1.359	0.942	ENSGALT00000016938	ENSGALG00000010402	HPGDS	18.967	30.772	73.991	39.090	-0.759
gga-miR-449b-5p	7.996	4.869	1.359	0.942	ENSGALT00000036373	ENSGALG00000047152	HBBA	70.218	115.320	220.605	246.446	-0.982
gga-miR-449b-5p	7.996	4.869	1.359	0.942	ENSGALT00000083335	ENSGALG00000044866	-	0.810	2.307	5.233	2.594	-0.779
gga-miR-449b-5p	7.996	4.869	1.359	0.942	ENSGALT00000039811	ENSGALG00000023772	HSPB7	0.365	2.101	10.688	2.971	-0.676
gga-miR-449b-5p	7.996	4.869	1.359	0.942	ENSGALT00000091351	ENSGALG00000049119	ZNF638	0.000	0.000	1.502	3.067	-0.851
gga-miR-449b-5p	7.996	4.869	1.359	0.942	ENSGALT00000049654	ENSGALG00000002377	ENO1	1.811	13.151	27.773	11.811	-0.748
gga-miR-449b-5p	7.996	4.869	1.359	0.942	ENSGALT00000063494	ENSGALG00000032588	ARPC1B	19.105	32.965	58.802	39.434	-0.852
gga-miR-449b-5p	7.996	4.869	1.359	0.942	ENSGALT00000045265	ENSGALG00000028520	CST3	192.429	208.400	465.314	285.714	-0.735
gga-miR-449b-5p	7.996	4.869	1.359	0.942	ENSGALT00000076652	ENSGALG00000038393	DMB2	10.035	19.697	26.477	18.703	-0.810
gga-miR-449b-5p	7.996	4.869	1.359	0.942	ENSGALT00000045095	ENSGALG00000048325	GBP	0.336	1.506	2.353	2.442	-0.993
gga-miR-449b-5p	7.996	4.869	1.359	0.942	ENSGALT00000054220	ENSGALG00000033694	IFI30	12.925	32.645	67.802	49.969	-0.933

gga-miR-449b-5p	7.996	4.869	1.359	0.942	ENSGALT00000000203	ENSGALG00000008022	TAPBP	16.005	27.456	34.611	31.539	-0.954
gga-miR-449b-5p	7.996	4.869	1.359	0.942	ENSGALT00000057383	ENSGALG00000026159	PDLIM7	0.097	0.465	1.677	0.768	-0.799
gga-miR-449b-5p	7.996	4.869	1.359	0.942	ENSGALT00000062132	ENSGALG00000043610	-	0.000	0.009	1.515	0.536	-0.736
gga-miR-449b-5p	7.996	4.869	1.359	0.942	ENSGALT00000081220	ENSGALG00000026152	-	4.957	10.320	14.275	12.658	-0.961
gga-miR-449b-5p	7.996	4.869	1.359	0.942	ENSGALT00000095657	ENSGALG00000043373	ARVCF	0.009	0.004	0.306	0.148	-0.793
gga-miR-449b-5p	7.996	4.869	1.359	0.942	ENSGALT00000103701	ENSGALG00000040484	SEC31A	0.000	0.000	1.304	0.231	-0.619
gga-miR-449b-5p	7.996	4.869	1.359	0.942	ENSGALT00000099340	ENSGALG00000013625	-	0.000	0.169	1.093	0.481	-0.805
gga-miR-449b-5p	7.996	4.869	1.359	0.942	ENSGALT00000003025	ENSGALG00000001956	OLFML3	6.726	9.117	17.147	10.572	-0.761
gga-miR-449b-5p	7.996	4.869	1.359	0.942	ENSGALT00000005163	ENSGALG00000003261	CAVIN1	84.002	117.874	239.929	130.746	-0.710
gga-miR-449b-5p	7.996	4.869	1.359	0.942	ENSGALT00000089897	ENSGALG00000045671	-	3.370	5.605	10.734	5.779	-0.725
gga-miR-449b-5p	7.996	4.869	1.359	0.942	ENSGALT00000038226	ENSGALG00000011865	SMTN	6.238	7.919	14.528	8.586	-0.698
gga-miR-449b-5p	7.996	4.869	1.359	0.942	ENSGALT00000012307	ENSGALG00000007611	RPL35A	223.206	297.753	521.852	494.983	-0.975
gga-miR-449b-5p	7.996	4.869	1.359	0.942	ENSGALT00000082983	ENSGALG00000035498	INPP5D	2.028	3.536	6.118	3.715	-0.782
gga-miR-449b-5p	7.996	4.869	1.359	0.942	ENSGALT00000100845	ENSGALG00000047024	ZNF469	0.414	0.686	1.566	0.886	-0.789
gga-miR-449b-5p	7.996	4.869	1.359	0.942	ENSGALT00000057614	ENSGALG00000037522	SBNO2	0.775	1.544	3.418	1.553	-0.700
gga-miR-449b-5p	7.996	4.869	1.359	0.942	ENSGALT00000060492	ENSGALG00000042534	NFATC1	0.083	0.850	1.128	0.558	-0.692
gga-miR-449b-5p	7.996	4.869	1.359	0.942	ENSGALT00000088232	ENSGALG00000045584	-	56.702	71.595	121.604	82.792	-0.784
gga-miR-449b-5p	7.996	4.869	1.359	0.942	ENSGALT00000079478	ENSGALG00000041380	BF2	55.194	85.931	129.764	103.967	-0.923
gga-miR-449b-5p	7.996	4.869	1.359	0.942	ENSGALT00000006250	ENSGALG00000003923	COL6A3	12.629	16.396	49.904	19.096	-0.617
gga-miR-449b-5p	7.996	4.869	1.359	0.942	ENSGALT00000096418	ENSGALG00000005473	PNAT3	0.000	0.894	0.886	2.538	-0.801
gga-miR-449b-5p	7.996	4.869	1.359	0.942	ENSGALT00000018516	ENSGALG00000011347	IHH	1.045	3.656	5.241	8.043	-0.939
gga-miR-449b-5p	7.996	4.869	1.359	0.942	ENSGALT00000092878	ENSGALG00000017302	SLCO2B1	1.549	2.803	5.689	4.409	-0.937
gga-miR-449b-5p	7.996	4.869	1.359	0.942	ENSGALT00000034529	ENSGALG00000021395	-	1.434	2.514	7.003	3.697	-0.786
gga-miR-449b-5p	7.996	4.869	1.359	0.942	ENSGALT00000101763	ENSGALG00000005582	KLHL18	0.001	0.001	0.271	0.001	-0.490
gga-miR-449b-5p	7.996	4.869	1.359	0.942	ENSGALT00000041362	ENSGALG00000024449	RAMP2	7.505	12.588	23.601	18.305	-0.931
gga-miR-449b-5p	7.996	4.869	1.359	0.942	ENSGALT00000088736	ENSGALG00000011276	BTG1	0.000	0.000	60.461	0.000	-0.490
gga-miR-449b-5p	7.996	4.869	1.359	0.942	ENSGALT00000040255	ENSGALG00000002160	B2M	148.224	227.047	409.309	282.319	-0.855
gga-miR-449b-5p	7.996	4.869	1.359	0.942	ENSGALT00000040078	ENSGALG00000023872	FAM129B	7.059	13.807	23.642	19.683	-0.962

gga-miR-449b-5p	7.996	4.869	1.359	0.942	ENSGALT00000077631	ENSGALG00000030602	ADAM33	3.397	5.729	11.781	6.890	-0.793
gga-miR-449b-5p	7.996	4.869	1.359	0.942	ENSGALT00000100966	ENSGALG00000023296	TAL1	1.101	1.504	5.733	4.472	-0.911
gga-miR-449b-5p	7.996	4.869	1.359	0.942	ENSGALT00000064544	ENSGALG00000042030	P4HA3	0.938	2.181	4.300	2.568	-0.833
gga-miR-449b-5p	7.996	4.869	1.359	0.942	ENSGALT00000020749	ENSGALG00000012710	NCS1	0.937	1.457	5.468	1.526	-0.583
gga-miR-449b-5p	7.996	4.869	1.359	0.942	ENSGALT00000053717	ENSGALG00000043829	-	0.348	0.775	1.683	1.559	-0.984
gga-miR-449b-5p	7.996	4.869	1.359	0.942	ENSGALT00000068153	ENSGALG00000040672	IGFBP4	15.151	21.315	41.074	32.272	-0.920
gga-miR-449b-5p	7.996	4.869	1.359	0.942	ENSGALT00000094753	ENSGALG00000015377	PHLDB2	0.014	0.007	0.858	1.557	-0.870
gga-miR-449b-5p	7.996	4.869	1.359	0.942	ENSGALT00000091454	ENSGALG00000051068	-	1.043	2.925	4.280	2.154	-0.667
gga-miR-449b-5p	7.996	4.869	1.359	0.942	ENSGALT00000055268	ENSGALG00000031546	-	215.197	317.034	466.343	307.179	-0.739
gga-miR-449b-5p	7.996	4.869	1.359	0.942	ENSGALT00000040657	ENSGALG00000024083	TCIM	9.301	13.030	27.010	17.649	-0.828
gga-miR-449b-5p	7.996	4.869	1.359	0.942	ENSGALT00000100490	ENSGALG00000053601	LPXN	0.000	0.017	0.199	0.000	-0.483
gga-miR-449b-5p	7.996	4.869	1.359	0.942	ENSGALT00000040832	ENSGALG00000006751	THY1	2.476	3.735	7.695	5.463	-0.884
gga-miR-449b-5p	7.996	4.869	1.359	0.942	ENSGALT00000035122	ENSGALG00000001573	P2RX1	1.958	3.910	7.806	3.874	-0.721
gga-miR-449b-5p	7.996	4.869	1.359	0.942	ENSGALT00000063990	ENSGALG00000005077	F8	0.270	0.623	1.079	0.670	-0.829
gga-miR-449b-5p	7.996	4.869	1.359	0.942	ENSGALT00000103928	ENSGALG00000047290	KIAA1755	1.162	1.718	3.847	2.770	-0.892
gga-miR-449b-5p	7.996	4.869	1.359	0.942	ENSGALT00000051952	ENSGALG00000030179	-	6.701	10.774	20.226	15.851	-0.932
gga-miR-449b-5p	7.996	4.869	1.359	0.942	ENSGALT00000095286	ENSGALG00000048409	TXNIP	16.251	22.318	47.768	52.353	-0.966
gga-miR-449b-5p	7.996	4.869	1.359	0.942	ENSGALT00000090049	ENSGALG00000000621	KANK3	14.965	19.711	34.857	24.111	-0.822
gga-miR-449b-5p	7.996	4.869	1.359	0.942	ENSGALT00000081517	ENSGALG00000001331	ST14	0.003	1.783	0.779	1.383	-0.507
gga-miR-449b-5p	7.996	4.869	1.359	0.942	ENSGALT00000094484	ENSGALG00000014155	OGDH	0.000	0.159	0.132	0.166	-0.808
gga-miR-449b-5p	7.996	4.869	1.359	0.942	ENSGALT00000025242	ENSGALG00000015656	-	2.177	2.823	7.271	4.561	-0.818
gga-miR-449b-5p	7.996	4.869	1.359	0.942	ENSGALT00000031378	ENSGALG00000019755	-	0.216	0.542	1.820	1.368	-0.933
gga-miR-30c-5p	5693.654	2767.107	1946.917	1879.616	ENSGALT00000097689	ENSGALG00000003774	ATP13A2	0.001	0.153	0.315	0.295	-0.956
gga-miR-449c-3p	3.178	1.483	0.362	0.472	ENSGALT00000084904	ENSGALG00000045602	REM1	3.791	5.906	12.033	6.206	-0.744
gga-miR-449c-3p	3.178	1.483	0.362	0.472	ENSGALT00000045095	ENSGALG00000048325	GBP	0.336	1.506	2.353	2.442	-0.996
gga-miR-449c-3p	3.178	1.483	0.362	0.472	ENSGALT00000095819	ENSGALG00000040241	NTRK3	0.000	0.780	1.302	0.000	-0.494
gga-miR-449c-3p	3.178	1.483	0.362	0.472	ENSGALT00000077338	ENSGALG00000030502	ADAMTS4	0.183	0.231	1.552	0.390	-0.618
gga-miR-449c-3p	3.178	1.483	0.362	0.472	ENSGALT00000040525	ENSGALG00000028409	SNRPG	0.000	11.821	10.060	0.000	-0.357

gga-miR-449c-3p	3.178	1.483	0.362	0.472	ENSGALT00000099821	ENSGALG00000047283	-	0.531	1.740	3.284	1.605	-0.817
gga-miR-449c-3p	3.178	1.483	0.362	0.472	ENSGALT00000004047	ENSGALG00000002562	ARAP3	1.645	2.328	5.511	2.972	-0.762
gga-miR-449c-3p	3.178	1.483	0.362	0.472	ENSGALT00000068230	ENSGALG00000028512	LCOR	0.000	0.713	0.396	0.314	-0.528
gga-miR-449c-3p	3.178	1.483	0.362	0.472	ENSGALT00000081220	ENSGALG00000026152	-	4.957	10.320	14.275	12.658	-0.992
gga-miR-449c-3p	3.178	1.483	0.362	0.472	ENSGALT00000011323	ENSGALG00000006992	MMP9	7.240	32.279	60.541	14.582	-0.623
gga-miR-449c-3p	3.178	1.483	0.362	0.472	ENSGALT00000023632	ENSGALG00000014662	RBP5	20.095	26.791	52.352	35.021	-0.832
gga-miR-449c-3p	3.178	1.483	0.362	0.472	ENSGALT00000034529	ENSGALG00000021395	-	1.434	2.514	7.003	3.697	-0.801
gga-miR-449c-3p	3.178	1.483	0.362	0.472	ENSGALT00000101763	ENSGALG00000005582	KLHL18	0.001	0.001	0.271	0.001	-0.517
gga-miR-449c-3p	3.178	1.483	0.362	0.472	ENSGALT00000057396	ENSGALG00000031325	TGFB1	5.522	8.927	14.237	9.964	-0.886
gga-miR-449c-3p	3.178	1.483	0.362	0.472	ENSGALT00000078625	ENSGALG00000042227	GNLY	16.877	31.484	45.924	33.285	-0.915
gga-miR-449c-3p	3.178	1.483	0.362	0.472	ENSGALT00000100282	ENSGALG00000013640	MBP	0.615	2.166	3.949	2.916	-0.961
gga-miR-449c-3p	3.178	1.483	0.362	0.472	ENSGALT00000102752	ENSGALG00000011680	EPHA5	0.000	0.688	0.370	0.387	-0.588
gga-miR-449c-3p	3.178	1.483	0.362	0.472	ENSGALT00000086266	ENSGALG00000010203	-	2.169	3.275	6.507	4.970	-0.911
gga-miR-449c-3p	3.178	1.483	0.362	0.472	ENSGALT00000060647	ENSGALG00000003521	TPM1	1.268	3.744	19.533	5.063	-0.671
gga-miR-449c-3p	3.178	1.483	0.362	0.472	ENSGALT00000105005	ENSGALG00000033497	FOXP4	0.000	0.011	0.435	0.009	-0.533
gga-miR-449c-3p	3.178	1.483	0.362	0.472	ENSGALT00000098007	ENSGALG00000030587	-	1.667	2.097	7.373	3.286	-0.705
gga-miR-449c-3p	3.178	1.483	0.362	0.472	ENSGALT00000091561	ENSGALG00000002510	IP6K1	0.000	0.274	0.524	0.350	-0.956
gga-miR-449c-3p	3.178	1.483	0.362	0.472	ENSGALT00000036371	ENSGALG00000028273	HBE1	0.000	0.044	0.581	0.216	-0.752
gga-miR-449c-3p	3.178	1.483	0.362	0.472	ENSGALT00000090049	ENSGALG00000000621	KANK3	14.965	19.711	34.857	24.111	-0.838
gga-miR-449c-3p	3.178	1.483	0.362	0.472	ENSGALT00000075410	ENSGALG00000003353	RAI14	0.957	1.126	6.569	2.892	-0.726
gga-miR-449c-3p	3.178	1.483	0.362	0.472	ENSGALT00000102497	ENSGALG00000004918	RAB9B	0.002	1.619	3.106	3.120	-0.994
gga-miR-449c-3p	3.178	1.483	0.362	0.472	ENSGALT00000081183	ENSGALG00000032220	ELN	1.706	5.807	8.190	5.272	-0.890
gga-miR-449c-3p	3.178	1.483	0.362	0.472	ENSGALT00000099432	ENSGALG00000042254	-	0.000	13.732	7.880	3.205	-0.401
gga-miR-449c-3p	3.178	1.483	0.362	0.472	ENSGALT00000040550	ENSGALG00000024034	PI4KB	0.011	0.014	3.142	1.409	-0.762
gga-miR-449c-3p	3.178	1.483	0.362	0.472	ENSGALT00000018516	ENSGALG00000011347	IHH	1.045	3.656	5.241	8.043	-0.896
gga-miR-449c-3p	3.178	1.483	0.362	0.472	ENSGALT00000068365	ENSGALG00000001749	ACSBG2	0.027	17.593	11.362	8.072	-0.587
gga-miR-449c-3p	3.178	1.483	0.362	0.472	ENSGALT00000062206	ENSGALG00000036498	EHD2	4.789	6.502	12.774	5.647	-0.610
gga-miR-449c-3p	3.178	1.483	0.362	0.472	ENSGALT00000091454	ENSGALG00000051068	-	1.043	2.925	4.280	2.154	-0.765

gga-miR-449c-3p	3.178	1.483	0.362	0.472	ENSGALT00000052260	ENSGALG00000037869	-	1.792	2.110	7.448	3.357	-0.697
gga-miR-449c-3p	3.178	1.483	0.362	0.472	ENSGALT00000059672	ENSGALG00000031094	EDA2R	3.582	5.661	9.884	6.899	-0.886
gga-miR-449c-3p	3.178	1.483	0.362	0.472	ENSGALT00000100490	ENSGALG00000053601	LPXN	0.000	0.017	0.199	0.000	-0.526
gga-miR-449c-3p	3.178	1.483	0.362	0.472	ENSGALT00000070640	ENSGALG00000040847	EDQM3	0.000	0.000	6.230	0.000	-0.517
gga-miR-449c-3p	3.178	1.483	0.362	0.472	ENSGALT00000026763	ENSGALG00000016580	SCARA5	0.801	2.072	4.507	2.277	-0.820
gga-miR-449c-3p	3.178	1.483	0.362	0.472	ENSGALT00000072324	ENSGALG00000037540	PLEKHO1	1.133	3.485	6.803	4.505	-0.926
gga-miR-449c-3p	3.178	1.483	0.362	0.472	ENSGALT00000075176	ENSGALG00000005204	-	0.000	0.000	0.763	0.000	-0.517
gga-miR-130b-5p	6.137	5.208	1.683	1.839	ENSGALT00000018516	ENSGALG00000011347	IHH	1.045	3.656	5.241	8.043	-0.883
gga-miR-130b-5p	6.137	5.208	1.683	1.839	ENSGALT00000022043	ENSGALG00000013537	TPM3	1.022	2.138	6.816	2.447	-0.731
gga-miR-130b-5p	6.137	5.208	1.683	1.839	ENSGALT00000038226	ENSGALG00000011865	SMTN	6.238	7.919	14.528	8.586	-0.756
gga-miR-130b-5p	6.137	5.208	1.683	1.839	ENSGALT00000043246	ENSGALG00000029024	TCF21	19.709	25.722	43.483	45.137	-0.996
gga-miR-130b-5p	6.137	5.208	1.683	1.839	ENSGALT00000068230	ENSGALG00000028512	LCOR	0.000	0.713	0.396	0.314	-0.165
gga-miR-130b-5p	6.137	5.208	1.683	1.839	ENSGALT00000064061	ENSGALG00000040055	-	0.073	1.134	2.727	1.717	-0.905
gga-miR-130b-5p	6.137	5.208	1.683	1.839	ENSGALT00000095657	ENSGALG00000043373	ARVCF	0.009	0.004	0.306	0.148	-0.889
gga-miR-130b-5p	6.137	5.208	1.683	1.839	ENSGALT00000102380	ENSGALG00000008725	KIF16B	0.000	0.101	0.264	0.000	-0.451
gga-miR-130b-5p	6.137	5.208	1.683	1.839	ENSGALT00000018446	ENSGALG00000011306	DES	27.266	73.695	266.779	88.525	-0.738
gga-miR-130b-5p	6.137	5.208	1.683	1.839	ENSGALT00000097062	ENSGALG00000004189	MAFK	0.051	0.709	1.377	0.358	-0.586
gga-miR-138-5p	11.765	6.384	2.718	2.661	ENSGALT00000000424	ENSGALG00000000318	CSRP1	28.182	48.476	112.192	58.500	-0.765
gga-miR-2954	138.000	85.274	40.809	38.254	ENSGALT00000065171	ENSGALG00000041634	ACTG2	2.957	19.227	100.269	23.366	-0.653
gga-miR-2954	138.000	85.274	40.809	38.254	ENSGALT00000093973	ENSGALG00000054718	FLNA	5.301	8.420	13.442	5.810	-0.519
gga-miR-2954	138.000	85.274	40.809	38.254	ENSGALT00000105703	ENSGALG00000040351	ACAP1	0.000	0.173	0.189	0.223	-0.943
gga-miR-2954	138.000	85.274	40.809	38.254	ENSGALT00000107116	ENSGALG00000003601	ATP2B4	0.000	0.662	1.826	1.436	-0.968
gga-miR-2954	138.000	85.274	40.809	38.254	ENSGALT00000075592	ENSGALG00000038399	PLEKHA6	0.004	0.032	0.506	0.292	-0.838
gga-miR-2954	138.000	85.274	40.809	38.254	ENSGALT00000094904	ENSGALG00000054971	ARHGAP30	1.304	2.328	3.862	2.583	-0.857
gga-miR-2954	138.000	85.274	40.809	38.254	ENSGALT00000080565	ENSGALG00000038458	LOXL1	0.582	1.410	4.554	0.458	-0.466
gga-miR-2954	138.000	85.274	40.809	38.254	ENSGALT00000046726	ENSGALG00000041266	CNN1	0.642	3.650	53.923	8.519	-0.601
gga-miR-2954	138.000	85.274	40.809	38.254	ENSGALT00000057614	ENSGALG00000037522	SBNO2	0.775	1.544	3.418	1.553	-0.718
gga-miR-2954	138.000	85.274	40.809	38.254	ENSGALT00000095286	ENSGALG00000048409	TXNIP	16.251	22.318	47.768	52.353	-0.940

gga-miR-2954	138.000	85.274	40.809	38.254	ENSGALT00000090049	ENSGALG00000000621	KANK3	14.965	19.711	34.857	24.111	-0.826
gga-miR-2954	138.000	85.274	40.809	38.254	ENSGALT00000095819	ENSGALG00000040241	NTRK3	0.000	0.780	1.302	0.000	-0.421
gga-miR-2954	138.000	85.274	40.809	38.254	ENSGALT00000080170	ENSGALG00000043754	GLUL	14.719	19.120	32.486	28.335	-0.943
gga-miR-2954	138.000	85.274	40.809	38.254	ENSGALT00000103185	ENSGALG00000025842	BRT-1	0.392	0.181	14.275	7.377	-0.791
gga-miR-2954	138.000	85.274	40.809	38.254	ENSGALT00000100475	ENSGALG00000040465	ZEB2	8.810	10.431	19.197	13.027	-0.784
gga-miR-2954	138.000	85.274	40.809	38.254	ENSGALT00000053904	ENSGALG00000005105	TPR	2.606	5.833	7.829	5.642	-0.870
gga-miR-2954	138.000	85.274	40.809	38.254	ENSGALT00000075454	ENSGALG00000040759	CPXM1	1.199	1.568	4.609	2.346	-0.734
gga-miR-2954	138.000	85.274	40.809	38.254	ENSGALT00000096902	ENSGALG00000055101	TASOR2	0.000	0.354	0.408	0.427	-0.947
gga-miR-2954	138.000	85.274	40.809	38.254	ENSGALT00000007813	ENSGALG00000004893	TMEM119	8.040	12.268	22.479	12.515	-0.729
gga-miR-2954	138.000	85.274	40.809	38.254	ENSGALT00000094484	ENSGALG00000014155	OGDH	0.000	0.159	0.132	0.166	-0.848
gga-miR-2954	138.000	85.274	40.809	38.254	ENSGALT00000097689	ENSGALG00000003774	ATP13A2	0.001	0.153	0.315	0.295	-0.996
gga-miR-2954	138.000	85.274	40.809	38.254	ENSGALT00000079478	ENSGALG00000041380	BF2	55.194	85.931	129.764	103.967	-0.933
gga-miR-2954	138.000	85.274	40.809	38.254	ENSGALT00000100966	ENSGALG00000023296	TAL1	1.101	1.504	5.733	4.472	-0.891
gga-miR-217-5p	8.117	8.851	0.819	2.460	ENSGALT00000099263	ENSGALG00000009587	GPHN	0.000	0.004	0.426	0.081	-0.821
gga-miR-217-5p	8.117	8.851	0.819	2.460	ENSGALT00000012260	ENSGALG00000007582	DPYSL3	9.796	14.033	20.795	17.596	-0.893
gga-miR-217-5p	8.117	8.851	0.819	2.460	ENSGALT00000100282	ENSGALG00000013640	MBP	0.615	2.166	3.949	2.916	-0.843
gga-miR-6660-3p	14.087	8.707	3.411	4.120	ENSGALT00000071222	ENSGALG00000041140	HTRA1	10.591	17.051	45.914	34.066	-0.930
gga-miR-6660-3p	14.087	8.707	3.411	4.120	ENSGALT00000076956	ENSGALG00000009655	AADAT	0.000	2.872	1.914	5.486	-0.703
gga-miR-6660-3p	14.087	8.707	3.411	4.120	ENSGALT00000036256	ENSGALG00000005678	FLNB	3.900	4.577	8.811	5.022	-0.724
gga-miR-6660-3p	14.087	8.707	3.411	4.120	ENSGALT00000095198	ENSGALG00000053511	APOLD1	3.515	5.991	10.967	5.670	-0.767
gga-miR-6660-3p	14.087	8.707	3.411	4.120	ENSGALT00000038637	ENSGALG00000009870	IL15	6.888	8.619	21.182	13.546	-0.852
gga-miR-6660-3p	14.087	8.707	3.411	4.120	ENSGALT00000099821	ENSGALG00000047283	-	0.531	1.740	3.284	1.605	-0.825
gga-miR-6660-3p	14.087	8.707	3.411	4.120	ENSGALT00000048133	ENSGALG00000016289	DST	0.000	0.000	1.503	0.224	-0.659
gga-miR-6660-3p	14.087	8.707	3.411	4.120	ENSGALT00000023206	ENSGALG00000014365	RBPJ	0.005	0.021	1.159	0.022	-0.574
gga-miR-6660-3p	14.087	8.707	3.411	4.120	ENSGALT00000103185	ENSGALG00000025842	BRT-1	0.392	0.181	14.275	7.377	-0.830
gga-miR-6660-3p	14.087	8.707	3.411	4.120	ENSGALT00000102330	ENSGALG00000029536	VPS11	0.003	0.004	5.461	0.002	-0.564
gga-miR-6660-3p	14.087	8.707	3.411	4.120	ENSGALT00000000203	ENSGALG00000008022	TAPBP	16.005	27.456	34.611	31.539	-0.983
gga-miR-458a-3p	221.124	128.526	49.547	58.280	ENSGALT00000061435	ENSGALG00000032736	MGLL	5.969	8.026	20.631	12.696	-0.837

gga-miR-449c-5p	10.423	3.526	1.891	1.095	ENSGALT00000034529	ENSGALG00000021395	-	1.434	2.514	7.003	3.697	-0.680
gga-miR-449c-5p	10.423	3.526	1.891	1.095	ENSGALT00000101763	ENSGALG00000005582	KLHL18	0.001	0.001	0.271	0.001	-0.368
gga-miR-449c-5p	10.423	3.526	1.891	1.095	ENSGALT00000092878	ENSGALG00000017302	SLCO2B1	1.549	2.803	5.689	4.409	-0.847
gga-miR-449c-5p	10.423	3.526	1.891	1.095	ENSGALT00000041362	ENSGALG00000024449	RAMP2	7.505	12.588	23.601	18.305	-0.846
gga-miR-449c-5p	10.423	3.526	1.891	1.095	ENSGALT00000088736	ENSGALG00000011276	BTG1	0.000	0.000	60.461	0.000	-0.368
gga-miR-449c-5p	10.423	3.526	1.891	1.095	ENSGALT00000040255	ENSGALG00000002160	B2M	148.224	227.047	409.309	282.319	-0.777
gga-miR-449c-5p	10.423	3.526	1.891	1.095	ENSGALT00000053717	ENSGALG00000043829	-	0.348	0.775	1.683	1.559	-0.889
gga-miR-449c-5p	10.423	3.526	1.891	1.095	ENSGALT00000064544	ENSGALG00000042030	P4HA3	0.938	2.181	4.300	2.568	-0.784
gga-miR-449c-5p	10.423	3.526	1.891	1.095	ENSGALT00000020749	ENSGALG00000012710	NCS1	0.937	1.457	5.468	1.526	-0.480
gga-miR-449c-5p	10.423	3.526	1.891	1.095	ENSGALT00000091454	ENSGALG00000051068	-	1.043	2.925	4.280	2.154	-0.715
gga-miR-449c-5p	10.423	3.526	1.891	1.095	ENSGALT00000100490	ENSGALG00000053601	LPXN	0.000	0.017	0.199	0.000	-0.387
gga-miR-449c-5p	10.423	3.526	1.891	1.095	ENSGALT00000055268	ENSGALG00000031546	-	215.197	317.034	466.343	307.179	-0.717
gga-miR-449c-5p	10.423	3.526	1.891	1.095	ENSGALT00000040657	ENSGALG00000024083	TCIM	9.301	13.030	27.010	17.649	-0.722
gga-miR-449c-5p	10.423	3.526	1.891	1.095	ENSGALT00000003855	ENSGALG00000025945	AVD	4.903	28.783	146.481	13.494	-0.446
gga-miR-449c-5p	10.423	3.526	1.891	1.095	ENSGALT00000068153	ENSGALG00000040672	IGFBP4	15.151	21.315	41.074	32.272	-0.810
gga-miR-449c-5p	10.423	3.526	1.891	1.095	ENSGALT00000022669	ENSGALG00000013993	VNN1	10.548	13.294	27.842	20.720	-0.750
gga-miR-449c-5p	10.423	3.526	1.891	1.095	ENSGALT00000059778	ENSGALG00000002335	DOCK3	0.000	0.000	0.357	0.121	-0.567
gga-miR-449c-5p	10.423	3.526	1.891	1.095	ENSGALT00000063990	ENSGALG00000005077	F8	0.270	0.623	1.079	0.670	-0.806
gga-miR-449c-5p	10.423	3.526	1.891	1.095	ENSGALT00000075176	ENSGALG00000005204	-	0.000	0.000	0.763	0.000	-0.368
gga-miR-449c-5p	10.423	3.526	1.891	1.095	ENSGALT00000095034	ENSGALG00000013031	ADA2	0.000	0.000	1.128	0.695	-0.682
gga-miR-449c-5p	10.423	3.526	1.891	1.095	ENSGALT00000099703	ENSGALG00000015573	FHL5	2.929	4.503	8.192	9.779	-0.863
gga-miR-449c-5p	10.423	3.526	1.891	1.095	ENSGALT00000095286	ENSGALG00000048409	TXNIP	16.251	22.318	47.768	52.353	-0.833
gga-miR-449c-5p	10.423	3.526	1.891	1.095	ENSGALT00000103928	ENSGALG00000047290	KIAA1755	1.162	1.718	3.847	2.770	-0.776
gga-miR-449c-5p	10.423	3.526	1.891	1.095	ENSGALT00000025242	ENSGALG00000015656	-	2.177	2.823	7.271	4.561	-0.685
gga-miR-449c-5p	10.423	3.526	1.891	1.095	ENSGALT00000036373	ENSGALG00000047152	HBBA	70.218	115.320	220.605	246.446	-0.876
gga-miR-449c-5p	10.423	3.526	1.891	1.095	ENSGALT00000040533	ENSGALG00000004594	Ii	89.087	177.920	287.842	212.679	-0.872
gga-miR-449c-5p	10.423	3.526	1.891	1.095	ENSGALT00000039811	ENSGALG00000023772	HSPB7	0.365	2.101	10.688	2.971	-0.577
gga-miR-449c-5p	10.423	3.526	1.891	1.095	ENSGALT00000049654	ENSGALG00000002377	ENO1	1.811	13.151	27.773	11.811	-0.735

gga-miR-449c-5p	10.423	3.526	1.891	1.095	ENSGALT00000091351	ENSGALG00000049119	ZNF638	0.000	0.000	1.502	3.067	-0.704
gga-miR-449c-5p	10.423	3.526	1.891	1.095	ENSGALT00000022043	ENSGALG00000013537	TPM3	1.022	2.138	6.816	2.447	-0.580
gga-miR-449c-5p	10.423	3.526	1.891	1.095	ENSGALT00000083335	ENSGALG00000044866	-	0.810	2.307	5.233	2.594	-0.726
gga-miR-449c-5p	10.423	3.526	1.891	1.095	ENSGALT00000045095	ENSGALG00000048325	GBP	0.336	1.506	2.353	2.442	-0.976
gga-miR-449c-5p	10.423	3.526	1.891	1.095	ENSGALT00000054220	ENSGALG00000033694	IFI30	12.925	32.645	67.802	49.969	-0.864
gga-miR-449c-5p	10.423	3.526	1.891	1.095	ENSGALT00000061435	ENSGALG00000032736	MGLL	5.969	8.026	20.631	12.696	-0.686
gga-miR-449c-5p	10.423	3.526	1.891	1.095	ENSGALT00000063494	ENSGALG00000032588	ARPC1B	19.105	32.965	58.802	39.434	-0.792
gga-miR-449c-5p	10.423	3.526	1.891	1.095	ENSGALT00000076652	ENSGALG00000038393	DMB2	10.035	19.697	26.477	18.703	-0.844
gga-miR-449c-5p	10.423	3.526	1.891	1.095	ENSGALT00000045265	ENSGALG00000028520	CST3	192.429	208.400	465.314	285.714	-0.592
gga-miR-449c-5p	10.423	3.526	1.891	1.095	ENSGALT00000081220	ENSGALG00000026152	-	4.957	10.320	14.275	12.658	-0.960
gga-miR-449c-5p	10.423	3.526	1.891	1.095	ENSGALT00000000203	ENSGALG00000008022	TAPBP	16.005	27.456	34.611	31.539	-0.968
gga-miR-449c-5p	10.423	3.526	1.891	1.095	ENSGALT00000057383	ENSGALG00000026159	PDLIM7	0.097	0.465	1.677	0.768	-0.705
gga-miR-449c-5p	10.423	3.526	1.891	1.095	ENSGALT00000103701	ENSGALG00000040484	SEC31A	0.000	0.000	1.304	0.231	-0.476
gga-miR-449c-5p	10.423	3.526	1.891	1.095	ENSGALT00000082983	ENSGALG00000035498	INPP5D	2.028	3.536	6.118	3.715	-0.740
gga-miR-449c-5p	10.423	3.526	1.891	1.095	ENSGALT00000038226	ENSGALG00000011865	SMTN	6.238	7.919	14.528	8.586	-0.608
gga-miR-449c-5p	10.423	3.526	1.891	1.095	ENSGALT00000003025	ENSGALG00000001956	OLFML3	6.726	9.117	17.147	10.572	-0.671
gga-miR-449c-5p	10.423	3.526	1.891	1.095	ENSGALT00000005163	ENSGALG00000003261	CAVIN1	84.002	117.874	239.929	130.746	-0.623
gga-miR-449c-5p	10.423	3.526	1.891	1.095	ENSGALT00000081507	ENSGALG00000035856	CD48	5.820	11.443	20.748	13.898	-0.816
gga-miR-449c-5p	10.423	3.526	1.891	1.095	ENSGALT00000089897	ENSGALG00000045671	-	3.370	5.605	10.734	5.779	-0.666
gga-miR-449c-5p	10.423	3.526	1.891	1.095	ENSGALT00000057614	ENSGALG00000037522	SBNO2	0.775	1.544	3.418	1.553	-0.640
gga-miR-449c-5p	10.423	3.526	1.891	1.095	ENSGALT00000100845	ENSGALG00000047024	ZNF469	0.414	0.686	1.566	0.886	-0.698
gga-miR-449c-5p	10.423	3.526	1.891	1.095	ENSGALT00000006250	ENSGALG00000003923	COL6A3	12.629	16.396	49.904	19.096	-0.505
gga-miR-449c-5p	10.423	3.526	1.891	1.095	ENSGALT00000018516	ENSGALG00000011347	IHH	1.045	3.656	5.241	8.043	-0.901
gga-miR-449c-5p	10.423	3.526	1.891	1.095	ENSGALT00000088232	ENSGALG00000045584	-	56.702	71.595	121.604	82.792	-0.691
gga-miR-449c-5p	10.423	3.526	1.891	1.095	ENSGALT00000039224	ENSGALG00000006775	TGM2	40.583	72.549	130.117	77.749	-0.738
gga-miR-449c-5p	10.423	3.526	1.891	1.095	ENSGALT00000079478	ENSGALG00000041380	BF2	55.194	85.931	129.764	103.967	-0.875
gga-miR-449d-5p	107.422	57.048	18.984	20.620	ENSGALT00000093583	ENSGALG00000031440	PTPRU	0.000	0.261	0.662	0.441	-0.947
gga-miR-449d-5p	107.422	57.048	18.984	20.620	ENSGALT00000068153	ENSGALG00000040672	IGFBP4	15.151	21.315	41.074	32.272	-0.916

gga-miR-449d-5p	107.422	57.048	18.984	20.620	ENSGALT00000054220	ENSGALG00000033694	IFI30	12.925	32.645	67.802	49.969	-0.945
gga-miR-449d-5p	107.422	57.048	18.984	20.620	ENSGALT00000055268	ENSGALG00000031546	-	215.197	317.034	466.343	307.179	-0.790
gga-miR-449d-5p	107.422	57.048	18.984	20.620	ENSGALT00000045095	ENSGALG00000048325	GBP	0.336	1.506	2.353	2.442	-0.999
gga-miR-449d-5p	107.422	57.048	18.984	20.620	ENSGALT00000034529	ENSGALG00000021395	-	1.434	2.514	7.003	3.697	-0.802
gga-miR-449d-5p	107.422	57.048	18.984	20.620	ENSGALT00000081517	ENSGALG00000001331	ST14	0.003	1.783	0.779	1.383	-0.581
gga-miR-449d-5p	107.422	57.048	18.984	20.620	ENSGALT00000000203	ENSGALG00000008022	TAPBP	16.005	27.456	34.611	31.539	-0.985
gga-miR-449d-5p	107.422	57.048	18.984	20.620	ENSGALT00000025242	ENSGALG00000015656	-	2.177	2.823	7.271	4.561	-0.818
gga-miR-6634-5p	2.092	1.515	0.135	0.786	ENSGALT00000091454	ENSGALG00000051068	-	1.043	2.925	4.280	2.154	-0.831
gga-miR-6634-5p	2.092	1.515	0.135	0.786	ENSGALT00000093100	ENSGALG00000003283	-	0.775	1.224	3.711	2.059	-0.967
gga-miR-6634-5p	2.092	1.515	0.135	0.786	ENSGALT00000046863	ENSGALG00000007186	TMEM268	1.214	3.264	7.684	4.670	-0.987
gga-miR-6634-5p	2.092	1.515	0.135	0.786	ENSGALT00000075592	ENSGALG00000038399	PLEKHA6	0.004	0.032	0.506	0.292	-0.972
gga-miR-6634-5p	2.092	1.515	0.135	0.786	ENSGALT00000050214	ENSGALG00000007623	CACNA1G	0.000	0.312	0.259	0.000	-0.326
gga-miR-6634-5p	2.092	1.515	0.135	0.786	ENSGALT00000011727	ENSGALG00000007247	PXN	0.001	1.756	3.481	0.823	-0.806
gga-miR-6634-5p	2.092	1.515	0.135	0.786	ENSGALT00000043563	ENSGALG00000026970	IFITM3	69.387	139.419	175.233	101.263	-0.763
gga-miR-6634-5p	2.092	1.515	0.135	0.786	ENSGALT00000038226	ENSGALG00000011865	SMTN	6.238	7.919	14.528	8.586	-0.913
gga-miR-6634-5p	2.092	1.515	0.135	0.786	ENSGALT00000022043	ENSGALG00000013537	TPM3	1.022	2.138	6.816	2.447	-0.999
gga-miR-6634-5p	2.092	1.515	0.135	0.786	ENSGALT00000086266	ENSGALG00000010203	-	2.169	3.275	6.507	4.970	-0.779
gga-miR-6634-5p	2.092	1.515	0.135	0.786	ENSGALT00000070640	ENSGALG00000040847	EDQM3	0.000	0.000	6.230	0.000	-0.862
gga-miR-6634-5p	2.092	1.515	0.135	0.786	ENSGALT00000039564	ENSGALG00000023689	ASS1	5.534	13.484	19.700	10.967	-0.961
gga-miR-6634-5p	2.092	1.515	0.135	0.786	ENSGALT00000020568	ENSGALG00000012594	NTRK2	0.519	1.008	2.902	1.500	-0.999
gga-miR-6634-5p	2.092	1.515	0.135	0.786	ENSGALT00000007990	ENSGALG00000004986	CERCAM	1.935	2.921	5.683	4.467	-0.922
gga-miR-6634-5p	2.092	1.515	0.135	0.786	ENSGALT00000030235	ENSGALG00000016785	IL1RL1	5.168	6.172	13.467	7.665	-0.952
gga-miR-6634-5p	2.092	1.515	0.135	0.786	ENSGALT00000103185	ENSGALG00000025842	BRT-1	0.392	0.181	14.275	7.377	-0.912
gga-miR-6634-5p	2.092	1.515	0.135	0.786	ENSGALT00000084904	ENSGALG00000045602	REM1	3.791	5.906	12.033	6.206	-0.929
gga-miR-6634-5p	2.092	1.515	0.135	0.786	ENSGALT00000054874	ENSGALG00000014784	MAST4	0.514	2.491	4.483	2.397	-0.990
gga-miR-6634-5p	2.092	1.515	0.135	0.786	ENSGALT00000044551	ENSGALG00000026891	NINJ1	4.369	5.886	12.100	8.629	-0.307
gga-miR-6634-5p	2.092	1.515	0.135	0.786	ENSGALT00000068230	ENSGALG00000028512	LCOR	0.000	0.713	0.396	0.314	-0.934
gga-miR-6634-5p	2.092	1.515	0.135	0.786	ENSGALT00000075454	ENSGALG00000040759	CPXM1	1.199	1.568	4.609	2.346	-0.913

gga-miR-6634-5p	2.092	1.515	0.135	0.786	ENSGALT00000100937	ENSGALG00000052892	-	0.280	1.030	4.678	1.507	-0.963
gga-miR-6634-5p	2.092	1.515	0.135	0.786	ENSGALT00000009457	ENSGALG00000005884	MAPKKK3L	7.455	8.910	20.971	13.386	-0.942
gga-miR-31-5p	1320.507	721.356	207.097	258.786	ENSGALT00000103036	ENSGALG00000051431	RPS15A	165.907	197.618	336.124	314.476	-0.847
gga-miR-31-5p	1320.507	721.356	207.097	258.786	ENSGALT00000010253	ENSGALG00000006343	ACTA2	19.882	58.427	157.816	79.771	-0.999
gga-miR-31-5p	1320.507	721.356	207.097	258.786	ENSGALT00000052239	ENSGALG00000039585	-	0.096	0.172	37.665	0.197	-0.543
gga-miR-31-5p	1320.507	721.356	207.097	258.786	ENSGALT00000050091	ENSGALG00000043582	-	3.010	5.985	41.582	51.534	-0.884
gga-miR-31-5p	1320.507	721.356	207.097	258.786	ENSGALT00000050748	ENSGALG00000041338	-	0.000	0.253	1.848	0.218	-0.629
gga-miR-7	23595.605	14786.197	2323.843	5333.079	ENSGALT00000023632	ENSGALG00000014662	RBP5	20.095	26.791	52.352	35.021	-0.909
gga-miR-301a-3p	4.210	1.039	0.984	1.142	ENSGALT00000102159	ENSGALG00000009512	TFPI2	166.715	149.873	393.079	239.139	-0.441
novel_147	1.353	0.569	0.087	0.000	ENSGALT00000036256	ENSGALG00000005678	FLNB	3.900	4.577	8.811	5.022	-0.624
novel_147	1.353	0.569	0.087	0.000	ENSGALT00000092705	ENSGALG00000007721	ARHGAP21	7.810	9.042	19.036	16.593	-0.874
novel_147	1.353	0.569	0.087	0.000	ENSGALT00000056798	ENSGALG00000042642	RARRES2	46.292	68.236	105.350	74.137	-0.817
gga-miR-490-3p	2.493	1.165	0.554	1.177	ENSGALT00000061435	ENSGALG00000032736	MGLL	5.969	8.026	20.631	12.696	-0.824
gga-miR-490-3p	2.493	1.165	0.554	1.177	ENSGALT00000083326	ENSGALG00000015908	COL12A1	20.394	20.084	48.874	22.602	-0.669
gga-miR-490-3p	2.493	1.165	0.554	1.177	ENSGALT00000107869	ENSGALG00000044996	TMEM71	0.000	0.000	0.576	0.523	-0.700
gga-miR-202-3p	29.032	17.149	6.251	9.523	ENSGALT00000092961	ENSGALG00000035923	MTMR11	0.681	1.143	2.456	1.468	-0.890
gga-miR-204	173.788	85.174	21.500	43.574	ENSGALT00000082443	ENSGALG00000037262	HOXC8	2.621	2.714	8.515	7.283	-0.850
gga-miR-204	173.788	85.174	21.500	43.574	ENSGALT00000093973	ENSGALG00000054718	FLNA	5.301	8.420	13.442	5.810	-0.653
gga-miR-204	173.788	85.174	21.500	43.574	ENSGALT00000041399	ENSGALG00000024486	-	9.139	16.489	30.695	14.348	-0.778
gga-miR-204	173.788	85.174	21.500	43.574	ENSGALT00000045637	ENSGALG00000028721	LMOD1	0.708	1.510	3.432	1.816	-0.868
gga-miR-204	173.788	85.174	21.500	43.574	ENSGALT00000055938	ENSGALG00000043675	ANTXR2	12.378	16.272	29.792	20.650	-0.881
gga-miR-204	173.788	85.174	21.500	43.574	ENSGALT00000068230	ENSGALG00000028512	LCOR	0.000	0.713	0.396	0.314	-0.547
gga-miR-204	173.788	85.174	21.500	43.574	ENSGALT00000096902	ENSGALG00000055101	TASOR2	0.000	0.354	0.408	0.427	-0.959
gga-miR-204	173.788	85.174	21.500	43.574	ENSGALT00000054723	ENSGALG00000035960	-	0.000	0.127	0.121	0.532	-0.568
gga-miR-449a	141.695	74.031	20.734	20.688	ENSGALT00000012307	ENSGALG00000007611	RPL35A	223.206	297.753	521.852	494.983	-0.955
gga-miR-449a	141.695	74.031	20.734	20.688	ENSGALT00000089897	ENSGALG00000045671	-	3.370	5.605	10.734	5.779	-0.755
gga-miR-449a	141.695	74.031	20.734	20.688	ENSGALT00000083335	ENSGALG00000044866	-	0.810	2.307	5.233	2.594	-0.808
gga-miR-449a	141.695	74.031	20.734	20.688	ENSGALT00000100490	ENSGALG00000053601	LPXN	0.000	0.017	0.199	0.000	-0.509

gga-miR-449a	141.695	74.031	20.734	20.688	ENSGALT00000103701	ENSGALG00000040484	SEC31A	0.000	0.000	1.304	0.231	-0.623
gga-miR-449a	141.695	74.031	20.734	20.688	ENSGALT00000081517	ENSGALG00000001331	ST14	0.003	1.783	0.779	1.383	-0.574
gga-miR-449a	141.695	74.031	20.734	20.688	ENSGALT00000088232	ENSGALG00000045584	-	56.702	71.595	121.604	82.792	-0.799
gga-miR-449a	141.695	74.031	20.734	20.688	ENSGALT00000000203	ENSGALG00000008022	TAPBP	16.005	27.456	34.611	31.539	-0.981
gga-miR-449a	141.695	74.031	20.734	20.688	ENSGALT00000057614	ENSGALG00000037522	SBNO2	0.775	1.544	3.418	1.553	-0.731
gga-miR-449a	141.695	74.031	20.734	20.688	ENSGALT00000095286	ENSGALG00000048409	TXNIP	16.251	22.318	47.768	52.353	-0.930
gga-miR-449a	141.695	74.031	20.734	20.688	ENSGALT00000100845	ENSGALG00000047024	ZNF469	0.414	0.686	1.566	0.886	-0.804
gga-miR-124a-3p	1.311	4.307	0.272	0.562	ENSGALT00000098007	ENSGALG00000030587	-	1.667	2.097	7.373	3.286	-0.568
gga-miR-124a-3p	1.311	4.307	0.272	0.562	ENSGALT00000095572	ENSGALG00000002225	RFFL	0.007	0.015	2.841	0.012	-0.482
gga-miR-124a-3p	1.311	4.307	0.272	0.562	ENSGALT00000014048	ENSGALG00000008621	NRP2	0.860	0.000	2.224	2.854	-0.870
gga-miR-135a-2-3p	1.719	1.246	0.271	0.581	ENSGALT00000084136	ENSGALG00000032868	AHCYL2	7.199	0.000	0.912	0.781	-0.702
gga-miR-449a	141.695	74.031	20.734	20.688	ENSGALT00000081028	ENSGALG00000042555	STAMBP	0.980	0.030	1.381	0.489	-0.053
gga-miR-449c-3p	3.178	1.483	0.362	0.472	ENSGALT00000011840	ENSGALG00000007319	BICDL1	0.043	0.000	0.208	0.099	-0.631
gga-miR-449c-3p	3.178	1.483	0.362	0.472	ENSGALT00000045144	ENSGALG00000052868	-	0.172	0.002	0.991	0.209	-0.510
gga-miR-449c-3p	3.178	1.483	0.362	0.472	ENSGALT00000102786	ENSGALG00000046708	PTGES	1.278	0.030	1.741	2.226	-0.396
gga-miR-449c-3p	3.178	1.483	0.362	0.472	ENSGALT00000099291	ENSGALG00000042900	GATAD2A	1.350	0.000	1.570	1.798	-0.245
gga-miR-6634-5p	2.092	1.515	0.135	0.786	ENSGALT00000105232	ENSGALG00000011405	SLC4A7	0.875	0.000	0.533	0.211	-0.241
gga-miR-6634-5p	2.092	1.515	0.135	0.786	ENSGALT00000024734	ENSGALG00000015324	-	37.089	0.000	8.837	0.000	-0.596
gga-miR-449d-5p	107.422	57.048	18.984	20.620	ENSGALT00000081028	ENSGALG00000042555	STAMBP	0.980	0.030	1.381	0.489	-0.050
gga-miR-449d-5p	107.422	57.048	18.984	20.620	ENSGALT00000063343	ENSGALG00000007767	DIS3L2	0.306	0.000	0.409	0.000	-0.159
gga-miR-449b-5p	7.996	4.869	1.359	0.942	ENSGALT00000071871	ENSGALG00000012952	BASP1	3.378	0.031	3.826	0.038	-0.235
gga-miR-449b-5p	7.996	4.869	1.359	0.942	ENSGALT00000067478	ENSGALG00000031216	FLOT2	1.018	0.000	1.613	0.000	-0.045
gga-miR-449b-5p	7.996	4.869	1.359	0.942	ENSGALT00000045144	ENSGALG00000052868	-	0.172	0.002	0.991	0.209	-0.521
gga-miR-449b-5p	7.996	4.869	1.359	0.942	ENSGALT00000097384	ENSGALG00000033171	TGM4	1.124	0.040	3.208	0.416	-0.301
gga-miR-449b-5p	7.996	4.869	1.359	0.942	ENSGALT00000101136	ENSGALG00000040098	EEF1D	138.575	100.184	276.236	152.421	-0.549
gga-miR-449b-5p	7.996	4.869	1.359	0.942	ENSGALT00000081028	ENSGALG00000042555	STAMBP	0.980	0.030	1.381	0.489	-0.103
gga-miR-449b-5p	7.996	4.869	1.359	0.942	ENSGALT00000101427	ENSGALG00000002818	VAT1	46.717	0.000	14.056	24.515	-0.473

gga-miR-22-3p	3285.671	1923.811	1744.071	888.692	ENSGALT00000073257	ENSGALG00000031450	MYO7A	0.000	0.012	0.000	0.545	-0.726
gga-miR-22-3p	3285.671	1923.811	1744.071	888.692	ENSGALT00000013275	ENSGALG00000008166	TLR15	0.000	0.000	0.202	0.290	-0.813
gga-miR-22-3p	3285.671	1923.811	1744.071	888.692	ENSGALT00000099432	ENSGALG00000042254	-	0.000	13.732	7.880	3.205	-0.318
gga-miR-2954	138.000	85.274	40.809	38.254	ENSGALT00000079072	ENSGALG00000034855	HK2	0.005	0.000	0.293	4.691	-0.572
gga-miR-2954	138.000	85.274	40.809	38.254	ENSGALT00000101669	ENSGALG00000046896	COLGALT1	0.000	0.000	0.000	1.894	-0.531
gga-miR-31-3p	68.163	30.520	7.562	17.545	ENSGALT00000010179	ENSGALG00000006298	ATG4B	0.000	0.000	0.245	0.421	-0.696
gga-miR-205b	0.946	0.178	0.186	0.000	ENSGALT00000079581	ENSGALG00000040741	DNM2	0.000	0.000	1.990	1.934	-0.639
gga-miR-449c-3p	3.178	1.483	0.362	0.472	ENSGALT00000096003	ENSGALG00000005188	MINDY4	0.018	0.448	0.315	0.773	-0.763
gga-miR-449c-3p	3.178	1.483	0.362	0.472	ENSGALT00000093025	ENSGALG00000032776	WDR7	0.000	0.000	0.000	0.973	-0.461
gga-miR-449c-3p	3.178	1.483	0.362	0.472	ENSGALT00000061959	ENSGALG00000008095	KIF23	0.000	0.386	0.492	0.804	-0.909
gga-miR-449c-3p	3.178	1.483	0.362	0.472	ENSGALT00000092609	ENSGALG00000013039	BID	0.000	0.566	0.002	2.866	-0.473
gga-miR-449c-3p	3.178	1.483	0.362	0.472	ENSGALT00000098176	ENSGALG00000028830	-	0.000	0.171	0.126	0.236	-0.825
gga-miR-449c-3p	3.178	1.483	0.362	0.472	ENSGALT00000095537	ENSGALG00000028561	SZT2	0.000	0.646	0.000	0.497	-0.288
gga-miR-449c-3p	3.178	1.483	0.362	0.472	ENSGALT00000044036	ENSGALG00000026591	GNG12	0.024	4.138	0.054	4.992	-0.394
gga-miR-449c-3p	3.178	1.483	0.362	0.472	ENSGALT00000103998	ENSGALG00000034475	EIF3G	0.000	7.082	0.000	11.292	-0.431
gga-miR-129-1-3p	21.275	11.165	6.017	3.806	ENSGALT00000018134	ENSGALG00000011124	ARHGAP24	0.000	0.425	0.734	0.790	-0.997
gga-miR-129-1-3p	21.275	11.165	6.017	3.806	ENSGALT00000005664	ENSGALG00000003578	FN1	10.375	30.378	67.299	26.656	-0.637
gga-miR-129-1-3p	21.275	11.165	6.017	3.806	ENSGALT00000069344	ENSGALG00000028147	CTSK	18.029	33.469	97.324	101.585	-0.912
gga-miR-129-1-3p	21.275	11.165	6.017	3.806	ENSGALT00000084926	ENSGALG00000038536	SH3BGRL3	18.307	50.409	20.995	77.351	-0.604
gga-miR-205a	5.803	3.520	2.372	1.368	ENSGALT00000107116	ENSGALG00000003601	ATP2B4	0.000	0.662	1.826	1.436	-0.900
gga-miR-205a	5.803	3.520	2.372	1.368	ENSGALT00000046917	ENSGALG00000014881	FAM184A	0.063	1.396	2.566	2.484	-0.969
gga-miR-135a-5p	105.055	43.817	13.092	13.579	ENSGALT00000104026	ENSGALG00000011642	ANKRD17	0.000	0.000	0.014	0.549	-0.483
gga-miR-449d-5p	107.422	57.048	18.984	20.620	ENSGALT00000095537	ENSGALG00000028561	SZT2	0.000	0.646	0.000	0.497	-0.268
gga-miR-449d-5p	107.422	57.048	18.984	20.620	ENSGALT00000096003	ENSGALG00000005188	MINDY4	0.018	0.448	0.315	0.773	-0.766
gga-miR-449d-5p	107.422	57.048	18.984	20.620	ENSGALT00000068778	ENSGALG00000001561	MXRA8	0.000	0.000	0.000	11.345	-0.488
gga-miR-449d-5p	107.422	57.048	18.984	20.620	ENSGALT00000106020	ENSGALG00000001319	CAMK1G	0.000	0.000	0.124	0.197	-0.822
gga-miR-6660-3p	14.087	8.707	3.411	4.120	ENSGALT00000063648	ENSGALG00000033638	SLC26A7	0.010	0.018	0.258	0.567	-0.761
gga-miR-6660-3p	14.087	8.707	3.411	4.120	ENSGALT00000051175	ENSGALG00000015101	BNC2	0.000	0.000	0.000	0.504	-0.468

gga-miR-6660-3p	14.087	8.707	3.411	4.120	ENSGALT00000098176	ENSGALG00000028830	-	0.000	0.171	0.126	0.236	-0.780
gga-miR-6660-3p	14.087	8.707	3.411	4.120	ENSGALT00000025431	ENSGALG00000015768	ANKRD6	0.334	1.129	1.107	2.084	-0.791
gga-miR-6660-3p	14.087	8.707	3.411	4.120	ENSGALT00000095537	ENSGALG00000028561	SZT2	0.000	0.646	0.000	0.497	-0.200
gga-miR-6660-3p	14.087	8.707	3.411	4.120	ENSGALT00000100656	ENSGALG00000008385	PPIP5K1	0.000	0.753	0.044	0.924	-0.359
gga-miR-34b-5p	500.250	318.885	106.799	45.009	ENSGALT00000095537	ENSGALG00000028561	SZT2	0.000	0.646	0.000	0.497	-0.235
gga-miR-34b-5p	500.250	318.885	106.799	45.009	ENSGALT00000056664	ENSGALG00000035679	LMO1	0.005	2.518	0.634	2.896	-0.529
gga-miR-449b-5p	7.996	4.869	1.359	0.942	ENSGALT00000104556	ENSGALG00000000225	MGAT4C	0.007	0.063	0.352	0.298	-0.950
gga-miR-449b-5p	7.996	4.869	1.359	0.942	ENSGALT00000097466	ENSGALG00000000336	PPP1R12B	2.294	9.372	7.333	10.934	-0.783
gga-miR-449b-5p	7.996	4.869	1.359	0.942	ENSGALT00000077925	ENSGALG00000029408	BAZ1B	0.000	0.000	0.000	2.036	-0.574
gga-miR-449b-5p	7.996	4.869	1.359	0.942	ENSGALT00000098176	ENSGALG00000028830	-	0.000	0.171	0.126	0.236	-0.803
gga-miR-449b-5p	7.996	4.869	1.359	0.942	ENSGALT00000095537	ENSGALG00000028561	SZT2	0.000	0.646	0.000	0.497	-0.216
gga-miR-449b-5p	7.996	4.869	1.359	0.942	ENSGALT00000068374	ENSGALG00000041213	ARHGAP17	0.000	0.186	0.000	0.649	-0.542
gga-miR-449b-5p	7.996	4.869	1.359	0.942	ENSGALT00000103998	ENSGALG00000034475	EIF3G	0.000	7.082	0.000	11.292	-0.443
gga-miR-449b-5p	7.996	4.869	1.359	0.942	ENSGALT00000084092	ENSGALG00000008114	SFXN2	0.146	0.217	0.949	2.239	-0.813
gga-miR-449b-5p	7.996	4.869	1.359	0.942	ENSGALT00000106020	ENSGALG00000001319	CAMK1G	0.000	0.000	0.124	0.197	-0.893
gga-miR-449b-5p	7.996	4.869	1.359	0.942	ENSGALT00000022483	ENSGALG00000013861	TNFAIP3	0.000	0.000	0.490	0.651	-0.913
gga-miR-449c-5p	10.423	3.526	1.891	1.095	ENSGALT00000077925	ENSGALG00000029408	BAZ1B	0.000	0.000	0.000	2.036	-0.493
gga-miR-449c-5p	10.423	3.526	1.891	1.095	ENSGALT00000095704	ENSGALG00000006385	UBXN7	0.302	0.438	0.430	2.881	-0.534
gga-miR-449c-5p	10.423	3.526	1.891	1.095	ENSGALT00000097466	ENSGALG00000000336	PPP1R12B	2.294	9.372	7.333	10.934	-0.917
gga-miR-449c-5p	10.423	3.526	1.891	1.095	ENSGALT00000050088	ENSGALG00000040098	EEF1D	0.254	0.289	0.265	10.446	-0.494
gga-miR-449c-5p	10.423	3.526	1.891	1.095	ENSGALT00000022483	ENSGALG00000013861	TNFAIP3	0.000	0.000	0.490	0.651	-0.745
gga-miR-449c-5p	10.423	3.526	1.891	1.095	ENSGALT00000106020	ENSGALG00000001319	CAMK1G	0.000	0.000	0.124	0.197	-0.733
gga-miR-449c-5p	10.423	3.526	1.891	1.095	ENSGALT00000084092	ENSGALG00000008114	SFXN2	0.146	0.217	0.949	2.239	-0.687
gga-miR-449c-5p	10.423	3.526	1.891	1.095	ENSGALT00000068374	ENSGALG00000041213	ARHGAP17	0.000	0.186	0.000	0.649	-0.555
gga-miR-449c-5p	10.423	3.526	1.891	1.095	ENSGALT00000085567	ENSGALG00000045540	-	13.476	19.590	26.424	37.803	-0.835
gga-miR-449c-5p	10.423	3.526	1.891	1.095	ENSGALT00000095537	ENSGALG00000028561	SZT2	0.000	0.646	0.000	0.497	-0.472
gga-miR-449c-5p	10.423	3.526	1.891	1.095	ENSGALT00000098176	ENSGALG00000028830	-	0.000	0.171	0.126	0.236	-0.911
gga-miR-204	173.788	85.174	21.500	43.574	ENSGALT00000097466	ENSGALG00000000336	PPP1R12B	2.294	9.372	7.333	10.934	-0.783

gga-miR-34c-5p	232.276	150.278	42.141	18.492	ENSGALT00000099703	ENSGALG00000015573	FHL5	2.929	4.503	8.192	9.779	-0.985
gga-miR-34c-5p	232.276	150.278	42.141	18.492	ENSGALT00000094753	ENSGALG00000015377	PHLDB2	0.014	0.007	0.858	1.557	-0.901
gga-miR-34c-5p	232.276	150.278	42.141	18.492	ENSGALT00000084092	ENSGALG00000008114	SFXN2	0.146	0.217	0.949	2.239	-0.849
gga-miR-34c-5p	232.276	150.278	42.141	18.492	ENSGALT00000095537	ENSGALG00000028561	SZT2	0.000	0.646	0.000	0.497	-0.204
gga-miR-34c-5p	232.276	150.278	42.141	18.492	ENSGALT00000054220	ENSGALG00000033694	IFI30	12.925	32.645	67.802	49.969	-0.916
gga-miR-34c-5p	232.276	150.278	42.141	18.492	ENSGALT00000081517	ENSGALG00000001331	ST14	0.003	1.783	0.779	1.383	-0.478
gga-miR-31-5p	1320.507	721.356	207.097	258.786	ENSGALT00000022483	ENSGALG00000013861	TNFAIP3	0.000	0.000	0.490	0.651	-0.855
gga-miR-31-5p	1320.507	721.356	207.097	258.786	ENSGALT00000072687	ENSGALG00000013649	CNOT7	4.029	28.072	3.814	31.770	-0.336
gga-miR-31-5p	1320.507	721.356	207.097	258.786	ENSGALT00000085724	ENSGALG00000007753	POLL	0.466	1.791	1.837	2.731	-0.887
gga-miR-449a	141.695	74.031	20.734	20.688	ENSGALT00000085567	ENSGALG00000045540	-	13.476	19.590	26.424	37.803	-0.872
gga-miR-449a	141.695	74.031	20.734	20.688	ENSGALT00000101521	ENSGALG00000037787	MEF2D	0.411	0.000	0.012	0.369	-0.397
gga-miR-449a	141.695	74.031	20.734	20.688	ENSGALT00000030611	ENSGALG00000019322	TNFRSF13C	0.044	0.000	0.193	0.440	-0.707
gga-miR-34c-5p	232.276	150.278	42.141	18.492	ENSGALT00000030611	ENSGALG00000019322	TNFRSF13C	0.044	0.000	0.193	0.440	-0.821
gga-miR-130b-5p	6.137	5.208	1.683	1.839	ENSGALT00000092143	ENSGALG00000016427	ADAM17	0.315	0.000	0.273	0.346	-0.406
gga-miR-449b-5p	7.996	4.869	1.359	0.942	ENSGALT00000030611	ENSGALG00000019322	TNFRSF13C	0.044	0.000	0.193	0.440	-0.780
gga-miR-449b-5p	7.996	4.869	1.359	0.942	ENSGALT00000097681	ENSGALG00000006791	TTI1	0.064	0.000	0.036	0.216	-0.460
gga-miR-449b-5p	7.996	4.869	1.359	0.942	ENSGALT00000097087	ENSGALG00000040364	HERPUD2	1.370	0.055	0.061	1.684	-0.102
gga-miR-449b-5p	7.996	4.869	1.359	0.942	ENSGALT00000101521	ENSGALG00000037787	MEF2D	0.411	0.000	0.012	0.369	-0.293
gga-miR-449b-5p	7.996	4.869	1.359	0.942	ENSGALT00000048408	ENSGALG00000006895	CEP131	1.062	0.001	0.000	1.493	-0.028
gga-miR-133c-3p	16.707	15.505	33.699	9.452	ENSGALT00000107995	ENSGALG00000010538	TTC27	0.000	1.029	0.000	5.118	-0.677
gga-miR-133c-3p	16.707	15.505	33.699	9.452	ENSGALT00000048408	ENSGALG00000006895	CEP131	1.062	0.001	0.000	1.493	-0.689
gga-miR-143-5p	326.004	491.847	823.909	272.415	ENSGALT00000048408	ENSGALG00000006895	CEP131	1.062	0.001	0.000	1.493	-0.831
gga-miR-143-5p	326.004	491.847	823.909	272.415	ENSGALT00000065165	ENSGALG00000029268	CIAPIN1	13.763	17.016	4.715	19.491	-0.885
gga-miR-143-5p	326.004	491.847	823.909	272.415	ENSGALT00000100689	ENSGALG00000010733	CEP170	0.275	0.000	0.000	2.014	-0.632
gga-miR-143-5p	326.004	491.847	823.909	272.415	ENSGALT00000075255	ENSGALG00000023898	-	1.473	0.082	0.103	1.661	-0.832
gga-miR-143-5p	326.004	491.847	823.909	272.415	ENSGALT00000027488	ENSGALG00000017018	INTS6	2.543	0.274	0.000	1.508	-0.794
gga-miR-143-5p	326.004	491.847	823.909	272.415	ENSGALT00000095588	ENSGALG00000006704	VPS8	1.563	1.554	0.000	1.014	-0.779
gga-miR-143-5p	326.004	491.847	823.909	272.415	ENSGALT00000074641	ENSGALG00000006851	-	2.618	1.886	0.019	1.297	-0.775

gga-miR-143-5p	326.004	491.847	823.909	272.415	ENSGALT00000075579	ENSGALG00000010313	MAST2	0.000	0.000	0.000	2.482	-0.553
gga-miR-143-5p	326.004	491.847	823.909	272.415	ENSGALT00000095537	ENSGALG00000028561	SZT2	0.000	0.646	0.000	0.497	-0.375
gga-miR-143-5p	326.004	491.847	823.909	272.415	ENSGALT00000098331	ENSGALG00000041423	MAP7D3	0.990	0.799	0.000	1.642	-0.950
gga-miR-133a-3p	107.574	99.409	202.785	80.977	ENSGALT00000048408	ENSGALG00000006895	CEP131	1.062	0.001	0.000	1.493	-0.632
gga-miR-133a-3p	107.574	99.409	202.785	80.977	ENSGALT00000107995	ENSGALG00000010538	TTC27	0.000	1.029	0.000	5.118	-0.596
gga-miR-133b	4.414	2.254	7.236	1.939	ENSGALT00000048408	ENSGALG00000006895	CEP131	1.062	0.001	0.000	1.493	-0.457
gga-miR-210b-5p	11.042	11.594	22.074	7.613	ENSGALT00000087758	ENSGALG00000000652	ACOT7	0.639	1.106	0.000	2.724	-0.818
gga-miR-210b-5p	11.042	11.594	22.074	7.613	ENSGALT00000070461	ENSGALG00000004171	RNF111	0.509	0.298	0.000	0.301	-0.796

Note: The expression levels of miRNA and mRNA were presented by TPM and FFKM, respectively.

Table 2. The list of top 30 significantly enriched GO terms of all the target genes of DE miRNAs in ovary during the four development stages of hens (p<0.01).

GO_Accession	Description	Term_Type	_*p*Value	DEG Number	DEG Name
GO:0009607	response to biotic stimulus	biological process	0.0010151	19	TNFAIP3, RPS15A, B2M, GNLY, ACTA2, ANKRD17, ENO1, ADAM17, STMN1, RARRES2, CNOT7, TLR15, TGFB1, IL15, SBNO2, TXNIP, GNG12, RBPJ, AVD
GO:0022409	positive regulation of cell-cell adhesion	biological process	0.0010221	8	IL15, TGFB1, FLOT2, KIF26B, THY1, Ii, PTPRU, IHH
GO:0002478	antigen processing and presentation of exogenous peptide antigen	biological process	0.0010465	2	B2M, Ii
GO:0016050	vesicle organization	biological process	0.0010929	11	ANKRD27, RAB7A, MYO7A, ZEB2, VPS8, DNM2, STX17, CLTCL1, VPS11, SEC31A, MYO18A
GO:0045799	positive regulation of chromatin assembly or disassembly	biological process	0.0014442	2	TAL1, TPR
GO:0085029	extracellular matrix assembly	biological process	0.001581	4	RAMP2, IHH, PHL DB2, TGFB1
GO:0051707	response to other organism	biological process	0.001705	18	GNG12, IL15, SBNO2, AVD, TGFB1, RBPJ, ENO1, ACTA2, ANKRD17, GNLY, B2M, TNFAIP3, RPS15A, TLR15, RARRES2, CNOT7, STMN1, ADAM17
GO:0043207	response to external biotic stimulus	biological process	0.0017737	18	RPS15A, TNFAIP3, GNLY, B2M, ANKRD17, ACTA2, ENO1, ADAM17, STMN1, TLR15, CNOT7, RARRES2, TGFB1, SBNO2, IL15, GNG12, RBPJ, AVD
GO:0030029	actin filament-based process	biological process	0.0023839	22	TPM1, BRT-1, STMN1, TPM3, EPHA5, LMOD1, ARHGAP17, CNN1, KIF23, ARPC1B, FLNB, MYO18A, PHLDB2, MYO7A, DPYSL3, PDLIM7, KANK3, TGFB1, NTRK3, EHD2, CACNA1G, SMTN
GO:0030036	actin cytoskeleton organization	biological process	0.002487	20	TGFB1, KANK3, FLNB, PDLIM7, EHD2, NTRK3, SMTN, ARPC1B, PHLDB2, MYO18A, DPYSL3, CNN1, ARHGAP17, KIF23, TPM1, STMN1, BRT-1, EPHA5, LMOD1, TPM3

GO:0045621	positive regulation of lymphocyte differentiation	biological process	0.0031567	5	Ii, IHH, INPP5D, IL15, TGFB1
GO:1900122	positive regulation of receptor binding	biological process	0.0033402	2	B2M, MMP9
GO:0032740	positive regulation of interleukin-17 production	biological process	0.0033766	2	IL15, TGFB1
GO:0045785	positive regulation of cell adhesion	biological process	0.00355	11	FLOT2, KIF26B, FN1, IL15, TGFB1, PTPRU, Ii, IHH, TGM2, THY1, NINJ1
GO:0038127	ERBB signaling pathway	biological process	0.0039636	6	RAB7A, KIF16B, MMP9, RBPJ, TGFB1, ADAM17
GO:0006692	prostanoid metabolic process	biological process	0.004016	3	PTGES, Ii, HPGDS
GO:0015629	actin cytoskeleton	cellular component	7.32E-06	22	ADAM17, TPM1, HSPB7, LPXN, BRT-1, LMOD1, TPM3, INTS6, CSRP1, VPS11, FLNB, ACTA2, ARPC1B, MYO7A, ENSGALG00000035960, PXN, MYO18A, PDLIM7, FLOT2, SMTN, ARHGAP21, GNG12
GO:0042611	MHC protein complex	cellular component	0.00036081	3	DMB2, B2M, Ii
GO:0042641	actomyosin	cellular component	0.00090579	6	PXN, MYO18A, FLNB, CSRP1, TPM1, PDLIM7
GO:0005912	adherens junction	cellular component	0.0010839	13	CSRP1, DST, FLNB, PDLIM7, DES, FLOT2, ADAM17, FAM129B, ARVCF, LPXN, PXN, PHLDB2, EPHA5
GO:0005884	actin filament	cellular component	0.0012877	7	VPS11, BRT-1, PDLIM7, TPM1, SMTN, TPM3, GNG12
GO:0070161	anchoring junction	cellular component	0.0013467	13	EPHA5, PHLDB2, LPXN, PXN, FAM129B, ARVCF, ADAM17, FLOT2,DES, DST, FLNB, CSRP1, PDLIM7
GO:0005576	extracellular region	cellular component	0.0017811	33	EHD2, IL15, TGFB1, ST14, HBBA, CEP131, AVD, CST3, IHH, APOLD1, CPXM1, FN1, KIF23, COL6A3, NRTN, B2M, IGFBP4, OLFML3, CTSK, SEMA6B, ADA2, DIPK2A, ENSGALG00000003283, RNPEP, LOXL1, HTRA1, BF2, COL12A1,

					ADAMTS4, ENSGALG00000024486, RARRES2, MMP9, TFPI2
GO:0001725	stress fiber	cellular component	0.0020894	5	PXN, CSRP1, FLNB, PDLIM7, TPM1
GO:0032432	actin filament bundle	cellular component	0.0032743	5	PXN, TPM1, FLNB, CSRP1, PDLIM7
GO:0005833	hemoglobin complex	cellular component	0.0038487	2	HBE1, HBBA
GO:0019842	vitamin binding	molecular function	0.0011092	5	RBP5, AVD, P4HA3, OGDH, ENSGALG00000031546,
GO:0004364	glutathione transferase activity	molecular function	0.0028833	3	ENSGALG00000005204, HPGDS, PTGES
GO:0030550	acetylcholine receptor inhibitor activity	molecular function	0.0031805	2	ENSGALG00000039585, ENSGALG00000043582
GO:0005030	neurotrophin receptor activity	molecular function	0.0031919	2	NTRK3, NTRK2

Table 3. The list of the significant KEGG classification of target genes of DE miRNAs in ovary during the four development stages of hens (p<0.05).

Terms	Pathways	*p* Value	DEGs Number	Gene Name
gga04144	Endocytosis	0.008722	11	EHD2, AGAP3, RAB7A, ARAP3, STAMBP, TGFB1, BF2, DNM2, CLTCL1, ACAP1, ENSGALG00000047393
gga04672	Intestinal immune network for IgA production	0.00988	4	IL15, TNFRSF13C, DMB2, PIGR
gga01230	Biosynthesis of amino acids	0.017353	5	PRPS2, AADAT, ENO1, GLUL, ASS1
gga00310	Lysine degradation	0.02805	4	AADAT, OGDH, COLGALT1, ENSGALG00000006818
gga04261	Adrenergic signaling in cardiomyocytes	0.031907	7	CAMK2B, TPM1, BRT-1, TPM3, AKT2, ATP2B4, ENSGALG00000035960
gga04010	MAPK signaling pathway	0.039785	10	MAPKKK3L, NTRK2, STMN1, TGFB1, GNG12, FLNA, CACNA1G, FLNB, AKT2, NFATC1
gga00051	Fructose and mannose metabolism	0.048926	3	GMDS, PFKFB3, HK2
